# Influence of metabolic dysfunction-associated steatotic liver disease on antituberculosis drug-induced liver injury

**DOI:** 10.1097/MD.0000000000044078

**Published:** 2025-08-29

**Authors:** Yi Shen, Jianping Liu

**Affiliations:** a Department of Respiratory and Critical Care Medicine, Affiliated Hospital of Hangzhou Normal University, Hangzhou, Zhejiang, China; b Department of Tuberculosis, Affiliated Hospital of Hangzhou Normal University, Hangzhou, Zhejiang, China.

**Keywords:** antituberculosis drugs, drug-induced liver injury, metabolic dysfunction-associated steatotic liver disease, non-alcoholic fatty liver disease

## Abstract

The risk of antituberculosis drug-induced liver injury (AT-DILI) in patients with metabolic dysfunction-associated steatotic liver disease (MASLD) is not clear. The aim of this study was to investigate incidence and risk factors associated with AT-DILI in MASLD patients. Retrospectively, a total of 120 MASLD patients who received antituberculosis medication from December 2017 to March 2023 were reviewed, including 91 males and 29 females. The participants were categorized into 2 cohorts based on the presence or absence of liver injury. Risk factors for AT-DILI were analyzed using logistic regression analysis. Among the 120 patients with treatment of tuberculosis complicated with MASLD, 28 (23.3%) patients developed AT-DILI. The remaining 92 (76.7%) patients did not develop AT-DILI. In the group of patients with liver injury, there were 26 cases of mild liver injury, one case of moderate liver injury, and one case of acute liver failure. Additionally, there were 23 cases of hepatocellular injury, 3 cases of cholestasis, and 2 cases of mixed liver injury. AT-DILI was observed during antituberculosis treatment 30.4 ± 17.6 days after the treatment began. There were significant differences in age, body mass index (BMI), platelet count, total bilirubin, fibrosis-4 (FIB-4) between the liver injury group, and the non-liver injury group (*P* < .05 in all). There were no significant differences in gender, hemoglobin, albumin, alanine aminotransferase, aspartate aminotransferase, alkaline phosphatase, γ-glutamyltransferase, total cholesterol, triglyceride, combined hypertension, and combined diabetes mellitus between the liver injury group, and the non-liver injury group (*P* > .05 in all). By logistic regression analysis, low BMI and FIB-4 were a high-risk factor for liver injury. The incidence of AT-DILI was high in patients with pulmonary tuberculosis complicated with MASLD. Clinicians should focus on the risk of AT-DILI in patients with low BMI and elevated FIB-4 scores.

## 1. Introduction

During the course of antituberculosis treatment, various types of adverse drug reactions may arise. Among these reactions, antituberculosis drug-induced liver injury (AT-DILI) is the most common and harmful, and it is also one of the common causes of DILI in China. Transient aminotransferase elevation is a common manifestation of mild cases of DILI, while severe cases can lead to liver failure, which can be life-threatening. In some instances, patients are compelled to discontinue antituberculosis therapy, thereby compromising the efficacy of tuberculosis treatment. However, the pathogenesis of DILI remains unclear. Previous research has indicated that the presence of chronic hepatitis B, chronic hepatitis C, and nonalcoholic fatty liver disease (NAFLD) can contribute to an increased occurrence of AT-DILI.^[1–4]^ Due to successful management of hepatitis B and the rising prevalence of obesity, NAFLD has emerged as the predominant chronic liver disease in China. Research has indicated that individuals diagnosed with metabolic disorders, including insulin resistance, hypertension, obesity, and dyslipidemia, face a heightened susceptibility to AT-DILI.^[5]^ Recently, the American Association for the Study of the Liver Diseases, the European Association for the Study of the Liver, and the Latin American Association for the Study of the Liver have proposed replacing the term NAFLD with metabolic dysfunction-associated steatotic liver disease (MASLD) to more accurately represent the disease’s underlying pathogenesis. MASLD is currently the most common chronic liver disease in clinical settings, affecting approximately 25% of adults worldwide.^[6]^ It is evident that a significant number of tuberculosis patients experience MASLD. however, there is a scarcity of research examining the impact of MASLD on AT-DILI. Consequently, this study primarily focuses on investigating the influence of MASLD on AT-DILI resulting from tuberculosis.

## 2. Materials and methods

### 2.1. Study design and participants

The data of 120 patients with MASLD complicated with an treatment of tuberculosis in our hospital were collected from December 2017 to March 2023 were reviewed. Research approval was obtained from the Ethics Committee of the Hangzhou Normal University Affiliated Hospital (2024(E2)-ks-027). All patients were informed and signed an informed consent. Patients or the public were not involved in the design, or conduct, or reporting, or dissemination plans of our research.

*Inclusion criteria*: According to the Guidelines for Diagnosis and Treatment of Tuberculosis. Diagnostic criteria for tuberculosis cases refer to Health Industry Standards of the People’s Republic of China (diagnostic criteria for tuberculosis) (WS 288-2017). Treatment included an intensive phase of 4 drugs (isoniazid [INH], rifampicin [RIF], pyrazinamide, and ethambutol) for 2 months, followed by a continuation phase of RIF and INH for 4 months. The diagnostic criteria of MASLD refer to A multisociety Delphi consensus statement on new fatty liver disease nomenclature. MASLD was diagnosed by hepatic steatosis by imaging, along with one or more of the specified 5 cardiometabolic risk factors. At least 1 out of 5: body mass index (BMI) ≥ 23 kg/m^2^ OR waist circumference > 94 cm (M) 80 cm (F); fasting serum glucose ≥ 5.6 mmol/L OR 2-hour post-load glucose levels ≥ 7.8 mmol/L OR hemoglobin A1c ≥ 5.7% OR type 2 diabetes OR treatment for type 2 diabetes; blood pressure ≥ 130/85 mm Hg OR specific antihypertensive drug treatment; plasma triglycerides ≥ 1.70 mmol/L OR lipid lowering treatment; plasma HDL-cholesterol ≤ 1.0 mmol/L (M) and ≤1.3 mmol/L (F) OR lipid lowering treatment.^[7]^ It meets the diagnostic criteria of DILI in the 2019 Guidelines for the Diagnosis and Treatment of Antituberculosis Drug-Induced Liver Injury. Prior to initiating antituberculosis treatment, the diagnosis of MASLD was established and normal baseline hepatic function. The exclusion criteria encompassed patients presenting with tuberculosis in conjunction with viral hepatitis A, B, C, D, E, autoimmune liver disease, genetic metabolic liver disease, hepatic tuberculosis, and other related conditions. Additionally, patients with a prolonged history of alcohol consumption and those exhibiting abnormal basic liver function were also excluded.

The following data were collected from electronic medical records: age, sex, alcohol ingestion, BMI, previous history of tuberculosis, site of tuberculosis infection (lung or other), date of prescription for antituberculosis drugs, regimen of antituberculosis drugs, serial liver function tests conducted every 2 weeks, including measurements of aspartate aminotransferase, alanine aminotransferase, and total bilirubin, symptoms of hepatitis such as anorexia, nausea, vomiting, generalized weakness, abdominal discomfort, and jaundice, as well as comorbidities such as hypertension and diabetes mellitus. The observation time was 8 weeks, and the time of abnormal liver function was recorded.

The diagnostic criteria of DILI refer to the Guidelines for the Diagnosis and Treatment of Antituberculosis Drug-induced Liver Injury in 2019.^[8]^ See Tables S1 and S2, Supplemental Digital Content, https://links.lww.com/MD/P793 for details.

### 2.2. Statistical analyses

SPSS Version 25 (International Business Machines Corporation, Armonk) for Windows was used for all statistical analyses. The data is reported in either mean ± standard deviation format or as a numerical count (n) with corresponding percentage. To compare the baseline characteristics of the 2 groups of participants, an independent-samples *t* test was employed for continuous variables, while the Pearson Chi-square test or Fisher exact test was used for categorical variables. Logistic regression was performed to test the association between indicators and the occurrence of liver injury. Statistical significance was determined by *P* < .05.

## 3. Results

### 3.1. Baseline clinical characteristics of the study population

A total of 120 patients with pulmonary tuberculosis complicated with MASLD participated in the study, including 91 males and 29 females. The mean age was 53.5 ± 18.3, with 81 farmers, 8 workers, 14 employees, and 17 other professions (retired/unemployed/not working). The mean BMI was 24.7 ± 3.5 kg/m^2^.

### 3.2. Comparison of liver injury group and non-liver injury group of pulmonary tuberculosis complicated with MASLD

Among the 120 patients with treatment of tuberculosis complicated with MASLD, 28 (23.3%) patients developed AT-DILI. The remaining 92 (76.7%) patients did not develop AT-DILI. The mean age of participants in the liver injury group was 60.8 ± 15.4 years, while in the non-liver injury group it was 51.3 ± 18.6 years. This difference in age between the 2 groups was found to be statistically significant (*P* = .016). Similarly, the mean BMI of participants in the liver injury group was 23.1 ± 3.4, whereas in the non-liver injury group it was 25.2 ± 3.5. This difference in BMI between the 2 groups was also found to be statistically significant (*P* = .005). There were significant differences in platelet count, total bilirubin, fibrosis-4 (FIB-4) between the liver injury group and the non-liver injury group (*P* < .05 in all). There were no significant differences in gender, hemoglobin, albumin, alanine aminotransferase, aspartate aminotransferase, alkaline phosphatase, γ-glutamyltransferase, total cholesterol, triglyceride, combined hypertension, and combined diabetes mellitus between the liver injury group and the non-liver injury group (*P* > .05 in all). See Table 1 for details.

**Table 1:** Participant characteristics.

Characteristics	Liver injury group (n = 28)	Non-liver injury group (n = 92)	*P*-value
Age (yr)	60.8 ± 15.4	51.3 ± 18.6	.016
18–39	2 (7.1%)	31 (33.7%)	
40–59	12 (42.9%)	27 (29.3%)	
≥60	14 (50.0%)	34 (37.0%)	
Sex (n, %)			.260
Male	19 (67.9%)	72 (78.3%)	
Female	9 (32.1%)	20 (21.7%)	
BMI (kg/m²)	23.1 ± 3.4	25.2 ± 3.5	.005
<25.0	17 (60.7%)	43 (46.7%)	
25.0–29.9	10 (35.7%)	39 (42.4%)	
>30.0	1 (3.6%)	10 (10.9%)	
Site of tuberculosis infection (n, %)			.879
Pulmonary TB	10 (35.7%)	36 (39.1%)	
Extrapulmonary TB	6 (21.4%)	16 (17.4%)	
Both pulmonary and extrapulmonary TB	12 (42.9%)	40 (43.5%)	
Hb (g/L)	132.9 ± 19.7	134.8 ± 23.5	.707
PLT (10^9^/L)	214.5 ± 84.5	268.9 ± 87.9	.005
ALB (g/L)	37.3 ± 5.8	38.0 ± 6.1	.562
ALT (U/L)	28.3 ± 16.9	27.3 ± 14.2	.768
AST (U/L)	25.4 ± 13.8	25.2 ± 11.4	.932
ALP (U/L)	100.1 ± 38.9	104.2 ± 45.6	.668
GGT (U/L)	57.3 ± 47.4	57.9 ± 55.7	.954
TBIL (μmol/L)	19.7 ± 11.5	15.4 ± 8.8	.037
FIB-4	2.2 ± 2.9	1.1 ± 0.8	.001
TC (mmol/L)	4.3 ± 1.4	4.4 ± 1.1	.811
TG (mmol/L)	1.5 ± 0.7	1.6 ± 0.9	.587
Hypertension (n, %)	13 (46.4%)	35 (38.0%)	.428
Diabetes mellitus (n, %)	5 (17.9%)	27 (29.3%)	.229

ALB = albumin, ALP = alkaline phosphatase, AST = aspartate aminotransferase, BMI = body mass index, FIB-4 = fibrosis-4, GGT = γ-glutamyltransferase, Hb = hemoglobin, PLT = platelet count, TBIL = total bilirubin, TC = total cholesterol, TG = triglyceride.

### 3.3. Characteristics of liver injury in pulmonary tuberculosis complicated with MASLD

A total of 28 cases of pulmonary tuberculosis complicated with MASLD who suffered a liver injury, with 19 males and 9 females affected. The mean age of the affected individuals was 60.8 ± 15.4 years. In the group of patients with liver injury, there were 26 cases of mild liver injury, one case of moderate liver injury, and one case of acute liver failure. Additionally, there were 23 cases of hepatocellular injury, 3 cases of cholestasis, and 2 cases of mixed liver injury. AT-DILI was observed during antituberculosis treatment 30.4 ± 17.6 days after the treatment began. See Table 2 for details.

**Table 2:** Characteristics of liver injury in pulmonary tuberculosis complicated with MASLD.

Basic characteristics	Results
N	28
Gender (male/female)	19/9
Age (mean ± SD, years)	60.8 ± 15.4
Degree of liver injury	
Grade 1 (mild)	26
Grade 2 (moderate)	1
Grade 3 (severe)	0
Grade 4 (acute liver failure)	1
Types of liver injury	
Hepatocyte injury	23
Cholestasis type	3
Mixed	2
Time of abnormal liver function (mean ± SD, days)	30.4 ± 17.6

MASLD = metabolic dysfunction-associated steatotic liver disease, SD = standard deviation.

### 3.4. Analysis of risk factors of liver injury in pulmonary tuberculosis complicated with MASLD

Were further analyzed by multivariate logistic regression. The results of the multivariate logistic regression analysis indicated that low BMI and FIB-4 were identified as a significant risk factors for the occurrence of AT-DILI in patients diagnosed with pulmonary tuberculosis and MASLD. See Table 3 for details.

**Table 3:** Analysis of risk factors of liver injury in pulmonary tuberculosis complicated with MASLD.

Factors	β	SE	Wald	*P*	OR (95% CI)
Age	0.014	0.015	0.823	.364	1.014 (0.984–1.045)
BMI	−0.181	0.090	4.073	.044	0.834 (0.700–0.995)
PLT	0.000	0.004	0.002	.966	1.000 (0.992–1.008)
TBIL	0.042	0.028	2.176	.140	1.043 (0.986–1.102)
ALT	0.046	0.025	3.426	.064	1.047 (0.997–1.100)
AST	−0.091	0.043	4.497	.074	0.913 (0.840–0.993)
FIB-4	0.983	0.420	5.482	.019	2.672 (1.174–6.084)
TC	−0.086	0.206	0.172	.678	0.918 (0.613–1.375)
TG	−0.217	0.318	0.464	.496	0.805 (0.432–1.502)

AST = aspartate aminotransferase, BMI = body mass index, FIB-4= fibrosis-4, MASLD = metabolic dysfunction-associated steatotic liver disease, PLT = platelet count, TBIL = total bilirubin, TC = total cholesterol, TG = triglyceride.

## 4. Discussion

Tuberculosis is a serious disease caused by the bacterium *Mycobacterium tuberculosis* chronic respiratory infectious diseases, one of the top 10 causes of death worldwide, are a global public health problem. Antituberculosis therapy frequently necessitates an extended duration of treatment and the use of multiple drugs in combination, thereby resulting in a high prevalence of AT-DILI. Apart from the inherent hepatotoxicity associated with the drugs, various factors such as advanced age, alcoholism, malnutrition, severe tuberculosis, HIV infection, and underlying liver disease significantly contribute to the heightened susceptibility to liver injury. Previous studies have shown that both chronic hepatitis B and chronic hepatitis C can potentially elevate the risk of DILI in individuals undergoing antituberculosis treatment.^[4,9,10]^ Similarly, nonalcoholic fatty liver disease has been implicated in increasing the likelihood of liver damage induced by anti-TB drugs.^[1]^

Presently, MASLD stands as the prevailing chronic liver ailment in clinical, impacting around 25% of the global adult population. Previous studies have shown that the probability of developing AT-DILI in patients with tuberculosis combined with NAFLD is 22% to 32.1%.^[1,11–13]^ Fang et al conducted a study in China in which they found that the occurrence of AT-DILI in patients with pulmonary tuberculosis and coexisting fatty liver was 22%, a significantly higher rate compared to the 14.3% observed in patients without fatty liver.^[11]^ Similarly, Cao et al from China showed that the incidence of AT-DILI in patients with pulmonary tuberculosis and NAFLD was 24.56%.^[12]^ Another study from China found that the occurrence of AT-DILI in patients with pulmonary tuberculosis and fatty liver was 32.1%, suggesting that fatty liver is a notable risk factor for liver injury in this population.^[13]^ Liu et al conducted a retrospective study involving 104 patients who received initial treatment for tuberculosis complicated with NAFLD, and found that 24 (23%) of these patients developed DILI.^[1]^ However, there was a lack of research examining the consequences of MASLD on AT-DILI. This study represents the first investigation into the impact of MASLD on AT-DILI, revealing that 28 cases (23.3%) out of 120 patients with tuberculosis and MASLD experienced AT-DILI. The prevalence of AT-DILI in this study is notably higher compared to the reported rates of 3.8% to 9.8% in China,^[14,15]^ but it aligns with the findings of the aforementioned study. These findings indicate that underlying liver disease significantly elevates the susceptibility to AT-DILI.

In our study, patients who experienced hepatic toxicity exhibited mild to moderate hepatitis (grades 1 and 2). the type of liver injury was mostly hepatocyte type. This is consistent with previous studies. Those participants who encountered hepatic impairment during the course of treatment did not manifest severe symptoms and did not necessitate treatment interruption or discontinuation. Furthermore, the results of this study demonstrate that patients with liver injury were generally older than those without liver injury. It is widely acknowledged that advanced age constitutes a crucial risk factor for AT-DILI. This phenomenon may be attributed to the physiological deterioration of tissues and organs in the elderly, the notable decline in physiological autoregulation capacity, the reduction in liver blood flow, the decrease in liver cell and liver drug enzyme activity, and the impairment of antituberculosis drug metabolite processing capability.^[16]^ However, the logistic regression analysis in this study indicates that advanced age is not a significant risk factor, which may be influenced by the limited sample size and the presence of other confounding factors. In the current investigation, it was observed that patients with liver injury exhibited a lower BMI compared to those without liver injury. Logistic regression analysis revealed that low BMI served as a significant risk factor for liver injury. Our findings indicate that a low BMI is associated with an increased risk of AT-DILI, as BMI serves as an indicator of overall health, including nutritional status. Prior research has demonstrated that malnutrition or hypoproteinemia independently contribute to the risk of AT-DILI.^[17]^ Furthermore, malnutrition has been identified as an independent risk factor for mortality in tuberculosis patients.^[18]^ Compared to lean individuals without NAFLD, lean people with NAFLD were significantly more likely to be older and male and had higher comorbidities (i.e., diabetes, hyperlipidemia, hypertension, metabolic syndrome, chronic kidney disease, and cardiovascular disease). The presence of NAFLD in lean individuals was independently associated with increased risk of all-cause and cardiovascular mortality.^[19,20]^ In a small longitudinal study with a median follow-up of 8.4 years, lean participants with biopsy-confirmed NAFLD exhibited a higher risk of liver-related mortality compared to their overweight or obese counterparts with NAFLD. Notably, the lean NAFLD cohort demonstrated a greater prevalence of advanced fibrosis at baseline within this study.^[21]^ In alignment with the aforementioned findings, this study demonstrates that patients with lean MASLD exhibited an elevated risk of AT-DILI and a higher baseline FIB-4 coefficient, suggesting a more severe degree of fibrosis at baseline. However, it is noteworthy that several cross-sectional studies have reported a lower prevalence of advanced fibrosis and cirrhosis among lean NAFLD participants compared to their overweight or obese counterparts with NAFLD.^[22,23]^ These findings underscore that lean patients with MASLD are at risk for progressive liver disease irrespective of weight gain.^[24]^ This risk may be associated with the extent of baseline liver fibrosis. However, further research involving larger sample sizes is warranted to substantiate these observations.

So far, the mechanism by which MASLD increases liver injury with antituberculosis drugs is unclear. There are several possible explanations for this. First, patients with MASLD are subjected to oxidative stress, leading to heightened levels of cellular oxidants and lipid peroxidation, while simultaneously depleting antioxidants.^[25,26]^ Consequently, the administration of drugs such as INH, which generate intracellular oxidants during metabolism, renders individuals with metabolic disorders more vulnerable to liver injury compared to those without such disorders.^[27,28]^ Second, the development of MASLD can lead to mitochondrial dysfunction.^[29]^ Stressed mitochondria can trigger hepatic necrosis when damaged by extrinsic factors, such as antituberculosis drugs.^[30]^ It is worth noting that INH, pyrazinamide, and RIF are all recognized for their potential to induce mitochondrial toxicity.^[31]^ Third, metabolic disorders induce changes in the expression of hepatic transporters, which are pivotal in facilitating hepatocyte metabolic processes.^[28,32]^ Some research has demonstrated that the inhibition of the bile salt export pump, as well as the basolateral multidrug resistance-associated proteins (MRP) 2, MRP3, and MRP4, can induce hepatocyte cholestasis and contribute to the pathogenesis of DILI.^[5,32]^ Furthermore, the administration of RIF, rifapentin, rifabutin, and clofazimine can intensify the impairment of hepatic transporters, thereby resulting in liver injury.^[33,34]^

In summary, our study found that the incidence of AT-DILI was high in patients with pulmonary tuberculosis complicated with MASLD. Clinicians should focus on the risk of AT-DILI in patients with low BMI and elevated FIB-4 scores. This study is subject to certain limitations. Firstly, it is important to note that the study design was retrospective, which inherently restricts the ability to establish a causal relationship between MASLD and liver injury resulting from antituberculosis drugs. Secondly, the sample size employed in this study was relatively small, thereby impeding a comprehensive assessment of the potential impact on antituberculosis treatment and prognosis. Consequently, it is imperative to conduct further prospective studies encompassing a larger cohort of patients in order to gain a more comprehensive understanding of the effect of MASLD on liver injury induced by antituberculosis drugs.

## 5. Conclusion

The incidence of AT-DILI was high in patients with pulmonary tuberculosis complicated with MASLD. Clinicians should focus on the risk of AT-DILI in patients with low BMI and elevated FIB-4 scores.

## Acknowledgments

The authors acknowledge the patients and their families.

## Author contributions

**Conceptualization:** Jianping Liu.

**Data curation:** Yi Shen, Jianping Liu.

**Investigation:** Yi Shen.

**Software:** Yi Shen, Jianping Liu.

**Writing – original draft:** Yi Shen.

**Writing – review & editing:** Jianping Liu.

## Supplementary Material



## References

[1] Liu YH Guo Y Xu H Feng H Chen DY. Impact of non-alcoholic simple fatty liver disease on antituberculosis drug-induced liver injury. Infection Drug Resistance. 2021;14:3667–71. 34526786 10.2147/IDR.S326386 PMC8437261

[2] Chang TE Huang YS Chang CH Perng C-L Huang Y-H Hou M-C. The susceptibility of anti-tuberculosis drug-induced liver injury and chronic hepatitis C infection: a systematic review and meta-analysis. J Chin Med Assoc. 2018;81:111–8. 29198550 10.1016/j.jcma.2017.10.002

[3] Chen L Bao D Gu L. Co-infection with hepatitis B virus among tuberculosis patients is associated with poor outcomes during anti-tuberculosis treatment. BMC Infect Dis. 2018;18:295. 29970037 10.1186/s12879-018-3192-8 PMC6029116

[4] Kim WS Lee SS Lee CM. Hepatitis C and not Hepatitis B virus is a risk factor for anti-tuberculosis drug induced liver injury. BMC Infect Dis. 2016;16:50. 26833347 10.1186/s12879-016-1344-2 PMC4736472

[5] Lim J Kim JS Kim HW. Metabolic disorders are associated with drug-induced liver injury during antituberculosis treatment: a multicenter prospective observational cohort study in Korea. Open Forum Infect Dis. 2023;10:ofad422. 37654787 10.1093/ofid/ofad422 PMC10468151

[6] Zhang P Dong X Zhang W. Metabolic-associated fatty liver disease and the risk of cardiovascular disease. Clinics Res Hepatol Gastroenterol. 2023;47:102063. 10.1016/j.clinre.2022.102063 36494073

[7] Rinella ME Lazarus JV Ratziu V. A multisociety Delphi consensus statement on new fatty liver disease nomenclature. J Hepatol. 2023;79:1542–56. 37364790 10.1016/j.jhep.2023.06.003

[8] Tuberculosis Branch of Chinese Medical Association. Guidelines for the diagnosis and treatment of antituberculosis drug-induced liver injury. Chin J Tubercul Respir Dis. 2019;42:343–56.

[9] Zheng J Guo MH Peng HW Cai X-L Wu Y-L Peng X-E. The role of hepatitis B infection in anti-tuberculosis drug-induced liver injury: a meta-analysis of cohort studies. Epidemiol Infect. 2020;148:e290. 33222713 10.1017/S0950268820002861 PMC7770377

[10] Chou C Veracruz N Chitnis AS Wong RJ. Risk of drug-induced liver injury in chronic hepatitis B and tuberculosis co-infection: a systematic review and meta-analysis. J Viral Hepat. 2022;29:1107–14. 36138556 10.1111/jvh.13751

[11] Wang F Yang YF. Analysis of risk factors of drug induced liver injury caused by antituberculosis treatment. Pract Pharm Clin Remedies 2014;17:1326–8.

[12] Cao SP Yang L Gao LC Fu MJ. Liver injury of 182 primary pulmonary tuberculosis patients induced by antituberculosis drugs. Cent South Pharm 2015;13:1003–6.

[13] Chi X Qi Y Chen J Cai HF Li WF Song SB. Logistic regression analysis of clinical characteristics and risk factors of drug-induced liver injury induced by primary antituberculosis therapy. Shaanxi Med J 2019;48:67–70.

[14] Xu N Yang JX Yang J. Incidence and associated risk factors of antituberculosis drug-induced hepatotoxicity among hospitalised patients in Wuhan, China. Eur J Hospital Pharmacy. 2022;29:217–21. 10.1136/ejhpharm-2020-002433 PMC9251158 32989000

[15] Zhang T Du J Yin X. adverse events in treating smear-positive tuberculosis patients in China. Int J Environ Res Public Health. 2015;13:86. 26729141 10.3390/ijerph13010086 PMC4730477

[16] Lim WS Avery A Kon OM Dedicoat M. Anti-tuberculosis drug-induced liver injury. BMJ (Clin Res Ed). 2023;383:e074866. 10.1136/bmj-2023-074866 37890885

[17] Sun Q Zhang Q Gu J. Prevalence, risk factors, management, and treatment outcomes of first-line antituberculosis drug-induced liver injury: a prospective cohort study. Pharmacoepidemiol Drug Saf. 2016;25:908–17. 26935778 10.1002/pds.3988

[18] Lin HS Lin MS Chi CC Ye JJ Hsieh CC. Nutrition assessment and adverse outcomes in hospitalized patients with tuberculosis. J Clin Med. 2021;10:2702. 34207380 10.3390/jcm10122702 PMC8235651

[19] Huang S Bao Y Zhang N Niu R Tian L. Long-term outcomes in lean and non-lean NAFLD patients: a systematic review and meta-analysis. Endocrine. 2023;85:134–41. 10.1007/s12020-023-03351-5 37253855

[20] Golabi P Paik J Fukui N Locklear CT de Avilla L Younossi ZM. Patients with lean nonalcoholic fatty liver disease are metabolically abnormal and have a higher risk for mortality. Clin Diabetes. 2019;37:65–72. 30705499 10.2337/cd18-0026 PMC6336127

[21] Feldman A Wernly B Strebinger G. Liver-related mortality is increased in lean subjects with non-alcoholic fatty liver disease compared to overweight and obese subjects. J Gastrointest Liver Dis. 2021;30:366–73. 10.15403/jgld-3622 34375373

[22] Weinberg EM Trinh HN Firpi RJ. Lean Americans with nonalcoholic fatty liver disease have lower rates of cirrhosis and comorbid diseases. Clin Gastroenterol Hepatol. 2021;19:996–1008.e6. 32629123 10.1016/j.cgh.2020.06.066

[23] Fracanzani AL Petta S Lombardi R. Liver and cardiovascular damage in patients with lean nonalcoholic fatty liver disease, and association with visceral obesity. Clin Gastroenterol Hepatol. 2017;15:1604–11.e1. 28554682 10.1016/j.cgh.2017.04.045

[24] Long MT Noureddin M Lim JK. AGA clinical practice update: diagnosis and management of nonalcoholic fatty liver disease in lean individuals: expert review. Gastroenterology. 2022;163:764–74.e1. 35842345 10.1053/j.gastro.2022.06.023 PMC9398982

[25] Clare K Dillon JF Brennan PN. Reactive oxygen species and oxidative stress in the pathogenesis of MAFLD. J Clin Transl Hepatol. 2022;10:939–46. 36304513 10.14218/JCTH.2022.00067 PMC9547261

[26] LeFort KR Rungratanawanich W Song BJ. Contributing roles of mitochondrial dysfunction and hepatocyte apoptosis in liver diseases through oxidative stress, post-translational modifications, inflammation, and intestinal barrier dysfunction. Cell Mol Life Sci. 2024;81:34. 38214802 10.1007/s00018-023-05061-7 PMC10786752

[27] Yew WW Chang KC Chan DP. Oxidative stress and first-line antituberculosis drug-induced hepatotoxicity. Antimicrob Agents Chemother. 2018;62:e02637–17. 29784840 10.1128/AAC.02637-17 PMC6105810

[28] Chen M Suzuki A Borlak J Andrade RJ Lucena MI. Drug-induced liver injury: interactions between drug properties and host factors. J Hepatol. 2015;63:503–14. 25912521 10.1016/j.jhep.2015.04.016

[29] Mposhi A Cortés-Mancera F Heegsma J. Mitochondrial DNA methylation in metabolic associated fatty liver disease. Front Nutr. 2023;10:964337. 37305089 10.3389/fnut.2023.964337 PMC10249072

[30] Han D Dara L Win S. Regulation of drug-induced liver injury by signal transduction pathways: critical role of mitochondria. Trends Pharmacol Sci. 2013;34:243–53. 23453390 10.1016/j.tips.2013.01.009 PMC3622802

[31] Porceddu M Buron N Roussel C Labbe G Fromenty B Borgne-Sanchez A. Prediction of liver injury induced by chemicals in human with a multiparametric assay on isolated mouse liver mitochondria. Toxicol Sci. 2012;129:332–45. 22987451 10.1093/toxsci/kfs197 PMC3446843

[32] Donepudi AC Cheng Q Lu ZJ Cherrington NJ Slitt AL. Hepatic transporter expression in metabolic syndrome: phenotype, serum metabolic hormones, and transcription factor expression. Drug Metab Disposition. 2016;44:518–26. 10.1124/dmd.115.066779 PMC4810770 26847773

[33] Morgan RE Trauner M van Staden CJ. Interference with bile salt export pump function is a susceptibility factor for human liver injury in drug development. Toxicol Sci. 2010;118:485–500. 20829430 10.1093/toxsci/kfq269

[34] Morgan RE van Staden CJ Chen Y. A multifactorial approach to hepatobiliary transporter assessment enables improved therapeutic compound development. Toxicol Sci. 2013;136:216–41. 23956101 10.1093/toxsci/kft176

