# Supplementary material for: Influence of metabolic dysfunction-associated steatotic liver disease on antituberculosis drug-induced liver injury
Source: Medicine (Baltimore). 2025 Aug 29;104(35):e44078. doi: 10.1097/MD.0000000000044078 (PMC12401279; doi:10.1097/MD.0000000000044078)
Supplement: Supplementary file 1 [file medi-104-e44078-s001.docx]

Supplementary Table 1 Diagnostic criteria for types of liver injury.

| Types of liver injury | Hepatocyte injury type | Cholestasis type | Mixed type |
| --- | --- | --- | --- |
| Diagnostic criteria | ALT≥3×ULN, and R≥5 | ALP≥2×ULN, and R≤2 | ALT≥3×ULN, ALP≥2×ULN, and 2<R<5. |

Supplementary Table 2 Diagnostic criteria for Degree of liver injury

| Degree of liver injury | Grade 1  (Mild liver injury) | Grade 2  (Moderate liver injury) | Grade 3  (Severe liver injury) | Grade 4  （Acute liver failure） |
| --- | --- | --- | --- | --- |
| Diagnostic criteria | A recoverable increase of serum ALT, ALP, or both, TBIL < 2.5 ULN | Elevated serum ALT, ALP, or both, TBIL ≥ 2.5 ULN | Elevated serum ALT, ALP, or both, TBIL ≥ 5 ULN | Elevated serum ALT, ALP, or both, TBIL≥10 ULN (171μmol/L) or daily increase ≥10mg/L or 17.1μmol/L, INR≥2.0 or PTA <40% |
